# Revealing Serotonin Derivatives in Safflower Seed Meal as Potential Anti-Ulcerative Colitis Drugs: In Vitro and Computational Evidence

**DOI:** 10.3390/molecules30132886

**Published:** 2025-07-07

**Authors:** Liang Zhang, Md Hasan Ali, Chao Jiang, Furong Fan, Furong Zhu, Yating Lu, Mengwei Jia, Haipeng Yin, Jianwang Wei, Dongsen Wu, Shenghui Chu, Min Liu

**Affiliations:** Key Laboratory of Xinjiang Phytomedicine Resource and Utilisation, Ministry of Education, Institute for Safflower Industry Research, Pharmacy College, Collaborative Innovation Center for Efficient Safflower Production and Resource Utilization, Shihezi University, North 4th Road 221, Shihezi 832002, China; zhangliang@stu.shzu.edu.cn (L.Z.); hasan.yzu@gmail.com (M.H.A.); 13918049521@163.com (C.J.); f721572869@163.com (F.F.); zfr210921@163.com (F.Z.); 15979116392@163.com (Y.L.); jmw079@126.com (M.J.); 13565162388@163.com (H.Y.); w16650554611@163.com (J.W.); wudongsen@stu.shzu.edu.cn (D.W.)

**Keywords:** safflower seed meal, serotonin derivatives, ulcerative colitis, molecular dynamics simulation, network pharmacology, molecular docking

## Abstract

This study evaluated the in vitro anti-inflammatory activity of serotonin derivatives from safflower seed powder and elucidated their mechanism against ulcerative colitis using network pharmacology. Compounds were extracted and purified via silica gel column chromatography, Sephadex LH-20 and semi-preparative HPLC. Structural characterization employed NMR and UPLC-Q-TOF-MS/MS with literature comparisons. Anti-inflammatory efficacy was assessed in lipopolysaccharide (LPS)-induced RAW 264.7 macrophages. Network pharmacology predicted targets, molecular docking analyzed binding interactions and molecular dynamics simulations assessed complex stability. Eleven serotonin derivatives were isolated; N-trans-Feruloyl-3,5-dihydroxyindolin-2-one (**1**) and Bufoserotonin A (**2**) were identified in safflower seed meal for the first time. Compounds **1**, **3**–**7** and **10** significantly reduced inflammatory factors, with N-feruloyl serotonin (**4**, FS) showing the strongest activity. Mechanistic studies revealed FS targets key molecules (STAT3, EGFR, ESR1, PTGS2, NF-κB1, and JUN), modulating PI3K-Akt, MAPK and cancer-related pathways. Molecular dynamics simulations confirmed FS-EGFR complex stability. Thus, two novel derivatives were isolated and FS demonstrated significant anti-inflammatory and potential anti-ulcerative colitis effects through multi-target, multi-pathway synergy, providing a foundation for developing safflower seed meal therapeutics.

## 1. Introduction

Ulcerative colitis (UC) is a chronic, recurrent and nonspecific inflammatory bowel disease (IBD) with complex pathogenesis [1]. It is widely recognized that the development of ulcerative colitis (UC) is primarily due to compromised intestinal mucosal barrier function, which is affected by genetic predisposition, environmental influences, immune system dysfunction and changes in intestinal microbiota [2]. In a clinical setting, UC presents with manifestations including abdominal discomfort, diarrhea, the presence of blood in stools and weight reduction. In more severe instances, complications can encompass gastrointestinal hemorrhage, bowel obstruction and malignancy [3]. According to recent statistics, the global number of patients with UC reached approximately 5 million in 2023, with an increasing prevalence. While the prevalence of UC has long been high in developed countries, developing countries are also experiencing a rise in incidence rates due to economic development and lifestyle changes [4]. Current pharmacological interventions for UC encompass 5-aminosalicylic acid, corticosteroids, immunosuppressants and monoclonal antibodies. But these treatments are associated with limitations, such as the potential for recurrence upon discontinuation and adverse reactions [5,6]. Thus, it is of significant clinical importance to identify therapeutic agents that offer substantial efficacy while minimizing the side effects and toxicity.

Safflower (*Carthamus tinctorius* L.) is a plant that grows every year or every two years. It is part of the Asteraceae family and can grow 30 to 120 cm. It features terminal heads and yellow flowers that transition to a dark red hue upon blooming. Originating from the “crescent zone” along the eastern Mediterranean coast, safflower has a cultivation history of at least 4500 years, making it one of the world’s oldest crops [7]. It is currently cultivated in India, Mexico, the United States, Canada, China, Iran and over 60 other countries. Owing to its diverse geographical distribution, safflower is known by various names, including “Golrang”, “Kajireh”, “Kafesheh”, “Zaffer”, “Fake Saffron” and “Dyer’s Saffron” [8]. Safflower is highly regarded in traditional medicine for its ability to improve blood circulation, alleviate blood stasis and reduce pain. It is commonly used to address various conditions associated with blood stasis, such as amenorrhea, dysmenorrhea, sharp pains in the chest and sides, injuries from trauma, sores, swelling and discomfort [9]. Recent studies have recognized that safflower is rich in bioactive compounds, including flavonoids, alkaloids, poly-alkynes, fatty acids and steroids. This plant has shown considerable therapeutic promise in preventing and treating cardiovascular diseases, metabolic syndrome and tumors by improving blood circulation, reducing inflammation, neutralizing free radicals and modulating cell proliferation, among various other mechanisms. Its multi-targeted therapeutic properties have become a focal point for the research and development of new pharmaceuticals [10].

Safflower seed meal, the residual product of safflower seeds post oil extraction via mechanical pressing or solvent extraction, is notably rich in protein (approximately 18–25%), dietary fiber (35–45%) and various small molecules, including flavonoids, lignans, hydroxycinnamoyl serotonin and other active secondary metabolites [11,12]. Due to its optimal crude protein content and fiber characteristics, it has become a significant raw material for the nutritional management of cattle and sheep in animal husbandry, offering the potential to reduce feeding costs and regulate rumen fermentation. In the industrial sector, its high fiber content facilitates the production of edible-grade nanofibers through electrostatic spinning technology, which is extensively utilized in food processing, cell culture scaffolds and drug delivery systems [13,14]. Nevertheless, the current comprehensive utilization rate of safflower seed meal remains below 40%, with a substantial portion still being discarded, leading to considerable resource wastage and environmental damage [15].

Xinjiang is the primary region for safflower cultivation and possesses abundant resources [16]. Current research, both domestically and internationally, predominantly focuses on comprehensive analyses of safflower filaments and safflower seed oil. However, a substantial research gap remains in the development of safflower seed meal, a byproduct of oil processing. This gap has hindered the advancement of serotonin derivatives (average content of 0.38–1.24 mg/g), which hold significant medicinal value [17]. Furthermore, our research group has identified that safflower seed meal has a notable effect on ulcerative colitis [18]. Consequently, this study focuses on serotonin derivatives in safflower seed meal based on the above research background and the previous literature. From the ethanol extract of safflower seed meal, eleven serotonin derivatives were isolated, with compounds **1** and **2** being identified from safflower seed meal for the first time. Additionally, an in vitro inflammation model was established using LPS-induced RAW 246.7 cells and the anti-inflammatory activities of the compounds were assessed using the Griess method and RT-qPCR. The findings revealed that compound **4** exhibited a significant inhibitory effect on cellular inflammation at a low concentration, surpassing the effects of other similar compounds. Finally, potential anti-inflammatory targets and mechanisms of action were investigated using network pharmacology, molecular docking and molecular dynamics simulation.

## 2. Results

### 2.1. Structural Characterization of Serotonin Derivatives

Compound **1** was identified as a gray solid with a sodium adduct observed in the ESI-MS at an *m*/*z* of 407.122, indicating a molecular formula of C_20_H_20_N_2_O_6_. The characterization of this compound was further supported by an analysis of its ^1^H-NMR spectrum at a frequency of 600 MHz, which presented a notable trans double-bond signal at a chemical shift of δ_H_ = 7.37 (d, *J* = 15.6 Hz, ^1^H). This was complemented by a detailed array of aromatic hydrogen signals situated at δ_H_ values of 7.09 (d, *J* = 2.1 Hz, ^1^H), 6.99 (dd, *J* = 8.2, 2.1 Hz, ^1^H), 6.87 (d, *J* = 2.5 Hz, ^1^H), 6.76 (d, *J* = 8.2 Hz, ^1^H), 6.73 (d, *J* = 8.4 Hz, ^1^H), and 6.69 (dd, *J* = 8.4, 2.5 Hz, ^1^H). In addition to the aromatic signals, the high-field region of the spectrum exhibited a methoxy hydrogen signal at δ_H_ = 3.87 (s, ^3^H). This region also included signals corresponding to a hypomethyl hydrogen and a methylene hydrogen, observed at δ_H_ values of 3.59–3.56 (m, ^1^H) and 2.14 (t, *J* = 7.7 Hz, ^2^H), respectively. The ^13^C-NMR spectrum recorded at 151 MHz in MeOD provided insights into the carbon framework of the compound, revealing two carbonyl carbons at chemical shifts of δ_C_ = 181.70 (C-3) and multiple aromatic ring signals at 154.96 (C-18), 134.49 (C-2), 133.74 (C-5), 116.72 (C-4), 113.04 (C-1,19), and 112.00 (C-14) ppm. Notably, the feruloyl portion of the aromatic carbon signals was characterized by resonance at δ_C_ values of 169.12 (C-9), 149.63 (C-6,13), 127.59 (C-12), 123.43 (C-17), and 111.41 (C-11). Moreover, a distinct set of trans-feruloyl signals was documented, alongside the identification of a quaternary carbon signal at δ_C_ = 77.00 (C-7) and a methoxy signal at δ_C_ = 56.33 (C-28). Two aminoethyl signals were also detected at δ_C_ = 38.24 (C-8,15), corresponding to additional carbon centers noted at δ_C_ = 142.18 (C-10) and 118.15 (C-16). These comprehensive spectroscopic analyses collectively facilitated a robust characterization of compound **1**. The spectral data are consistent with those reported in the literature [19] for trans-feruloyl-3,5-dihydroxyindolin-2-one.

Compound **2** is characterized as a colorless amorphous powder, which primarily contributes to its distinct physical properties. The compound was analyzed using ESI-MS, where it exhibited an *m*/*z* of 220.1091 for the ion [M + H]^+^. This measurement is crucial in confirming the molecular weight of the compound, further allowing for its identification. The molecular formula for compound **2** is established as C_11_H_13_N_3_O_2_, which indicates that it comprises eleven carbon atoms, thirteen hydrogen atoms, three nitrogen atoms, and two oxygen atoms. This composition provides insight into the overall structure and potential reactivity of the compound. Additionally, nuclear magnetic resonance (NMR) spectroscopy was conducted at a frequency of 400 MHz in DMSO, revealing numerous distinct chemical shifts. For instance, the ^1^H-NMR spectrum features a singlet at δ_H_ 8.62 representing one hydrogen atom, alongside various doublets, a doublet of doublets, and triplet signals that showcase the complexity of the compound’s molecular environment. Notably, a triplet at δ_H_ 2.77 highlights the presence of additional hydrogen atoms, contributing to the characterization of the compound’s structure. The ^13^C-NMR spectrum, recorded at 101 MHz in DMSO, provides further valuable insight into the compound’s carbon framework. The spectral data show resonances at various chemical shifts, such as δ_C_ 150.19 for C-3 and 102.24 for C-2, which help in mapping out the carbon skeleton of the compound. Overall, the spectral data obtained from this analysis were consistent with the previously published literature [20], allowing for the definitive identification of compound **2** as Bufoserotonin A. This identification underscores the significance of the spectral analyses employed, as they confirm the identity of the compound and offer a framework for understanding its properties and potential applications.

**Figure 1:**
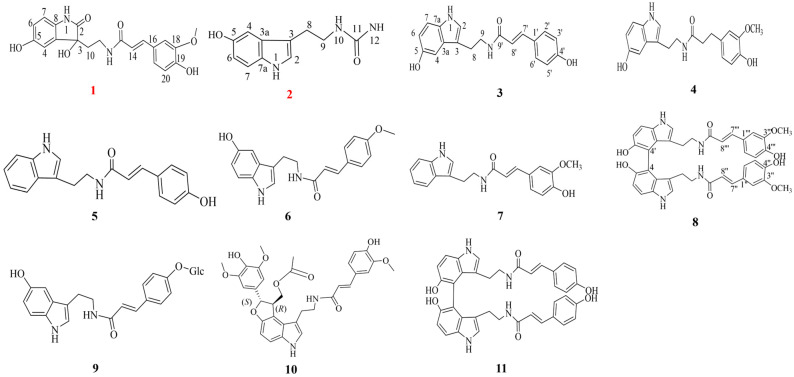
Serotonin derivatives isolated from safflower seed meal (**1**–**11**). The numbers added in the picture are used to indicate the atom numbering in each compound.

The nuclear magnetic resonance (NMR) spectra of compounds **1** and **2** are included in the Supplementary Materials (Figures S1–S8). In addition, nine known serotonin derivatives were identified by comparison with the reported literature as follows: N-(p-Coumaroyl)-serotonin (**3**) [17], N-Feruloyl serotonin (**4**) [21], N-(p-Coumaroyl)-tryptamine (**5**) [22], N-p-Methoxy cinnamoyl serotonin (**6**) [23], N-trans-feruloyl tryptamine (**7**) [23], 4,4″-bis(N-p-Feruloy)serotonin (**8**) [24], Tracheloside (**9**) [21], (±)-Carthatin A (**10**) [25] and 4,4″-bis(N-p-coumaroyl)serotonin (**11**) [24]. The structural formulas of compounds 1-11 are shown in Figure 1.

### 2.2. Effect of Serotonin Derivatives on Nitric Oxide (NO) Level

**Figure 2:**
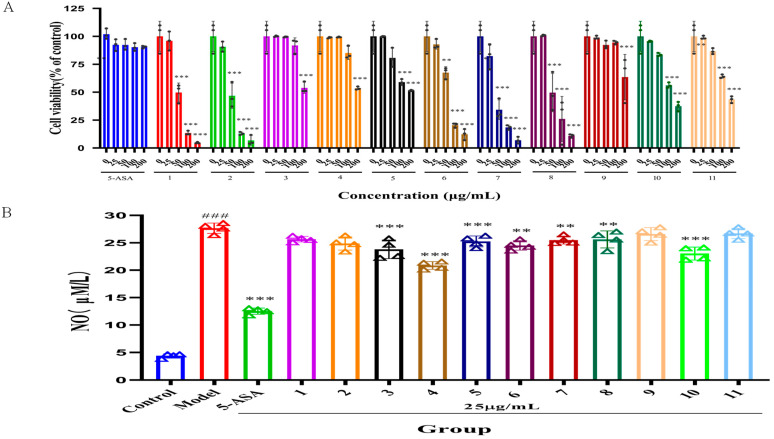
Impact of serotonin derivatives (**1**–**11**) on RAW 264.7 cell lines. (**A**) Influence of serotonin derivatives on viability of RAW 264.7 cells (n = 3). (**B**) Effect of serotonin derivatives on inhibition of NO production (n = 4). ^###^ *p* < 0.001 compared to control. ** *p* < 0.01, *** *p* < 0.001 in comparison to model group.

The activity of all monomer compounds isolated from safflower seed meal was investigated, with a specific focus on their anti-inflammatory properties. Initially, the impact of positive drugs and serotonin derivatives on the viability of RAW 264.7 cells was assessed through the CCK-8 assay, with the results presented in (Figure 2A). After a 24 h incubation period, different concentrations of 5-Aminosalicylic Acid (5-ASA) did not influence the viability of the RAW 264.7 cell line, maintaining over 90% viability (*p* > 0.05). Moreover, at a concentration of 25 μg/mL, serotonin derivatives **1**–**11** exhibited no significant impact on RAW 264.7 cell viability when compared to the control group (*p* > 0.05). Consequently, concentrations below 25 μg/mL were chosen for the follow-up experiments in this research.

The impact of a serotonin derivative (25 μg/mL) on NO levels in LPS-induced RAW 264.7 cells was assessed using the Griess method [26], with 5-ASA as a positive control. The findings are presented in (Figure 2B). The comparison with the control group revealed that the stimulation with 1 μg/mL LPS led to a significant increase in the levels of NO within the cells. This finding confirms that an effective in vitro inflammation model was successfully established. Furthermore, this study investigated the effects of various serotonin derivatives—specifically derivatives **3**, **4**, **5**, **6**, **7**, **8**, and 10—as well as 5-ASA. At a concentration of 25 μg/mL, these compounds demonstrated a significant ability to suppress NO secretion (*p* < 0.01, *p* < 0.001). Among the various compounds tested, serotonin derivative **4** stood out due to its pronounced anti-inflammatory effects, as it exhibited significant differences when compared to the model group (*p* < 0.001). Additionally, the study highlighted that among all the tested compounds, FS displayed the highest efficacy in inhibiting the production of NO, suggesting its potential as a leading candidate for further investigation in the context of inflammation-related therapies.

### 2.3. Effect of Serotonin Derivatives on Inflammatory Factors

**Figure 3:**
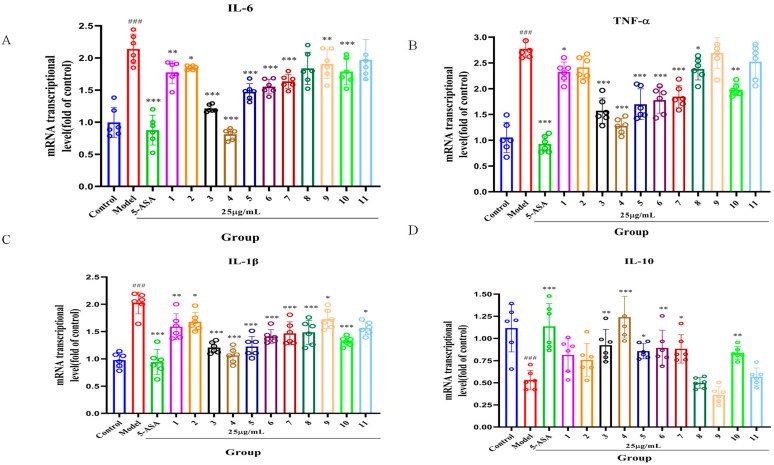
The impact of serotonin derivatives (**1**–**11**) on the inflammatory markers IL-6 (**A**), TNF-α (**B**), IL-1β (**C**), and IL-10 (**D**) in RAW 246.7 cells was assessed (n = 5). ^###^ *p* < 0.001 compared to the control group. * *p* < 0.05, ** *p* < 0.01, and *** *p* < 0.001 in comparison to the model group.

Inflammatory cytokines are integral to the pathogenesis and progression of ulcerative colitis [1]. To explore the effects of serotonin derivatives on inflammatory cytokines, we measured the mRNA expression levels of IL-6, TNF-α, IL-1β, and IL-10 utilizing RT-qPCR. As depicted in Figure 3, the model group displayed a significant increase in mRNA levels of IL-6, TNF-α, and IL-1β compared to the control group, along with a substantial reduction in IL-10 mRNA levels (*p* < 0.001). Both 5-ASA and serotonin derivatives **1**, **2**, **3**, **4**, **5**, **6**, **7**, **9**, and **10** were associated with a noteworthy decrease in mRNA levels of IL-6, TNF-α, and IL-1β when compared to the model group (*p* < 0.05, *p* < 0.01, *p* < 0.001). Furthermore, serotonin derivatives **3**, **4**, **5**, **6**, and **10** significantly improved IL-10 mRNA expression levels (*p* < 0.05, *p* < 0.01, and *p* < 0.001). Among the **11** derivatives analyzed, serotonin derivative **4** exhibited the most pronounced anti-inflammatory properties (*p* < 0.001).

### 2.4. Construction of Target Database

**Figure 4:**
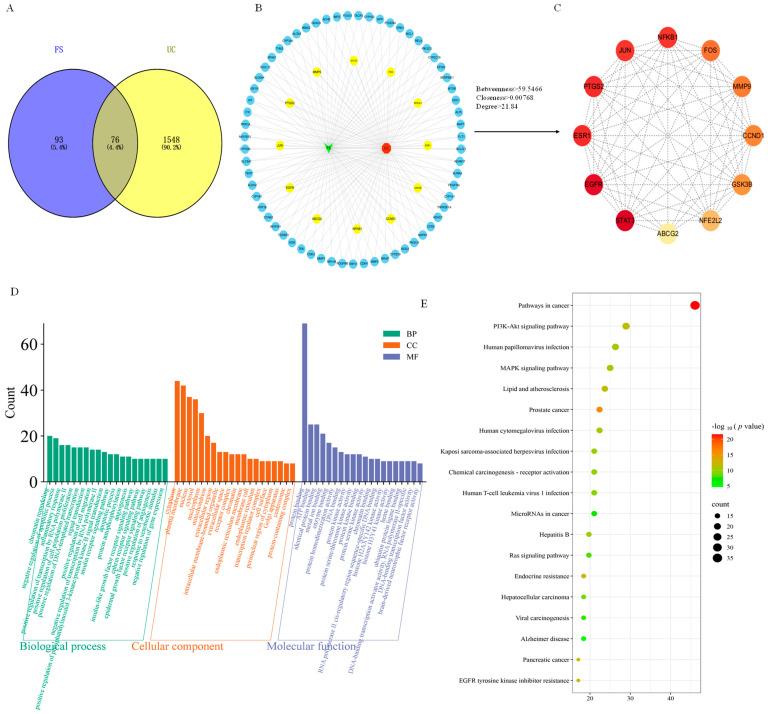
Network pharmacology analysis of FS treatment for UC. (**A**) Intersection of FS and UC targets. (**B**) Component–disease–potential target network of FS treatment for UC. (**C**) Core targets of FS treatment for UC. (**D**) GO enrichment analysis. (**E**) KEGG enrichment analysis.

A total of 169 FS targets were identified using the Swiss Target Prediction and Target Net databases. Additionally, 1624 targets related to ulcerative colitis were retrieved from the GeneCards and CTD online databases using the search term “Ulcerative Colitis”, with relevance scores exceeding 22.75 and 4.38, respectively, and duplicates were subsequently removed. The intersection of these datasets, visualized using a Venn diagram, revealed 76 potential targets (Figure 4A).

### 2.5. PPI Network

The 76 screened action targets were input into the STRING database for protein–protein interaction (PPI) network analysis. Subsequently, Cytoscape software was used to construct the component–target–disease interaction network graph, which comprised 76 nodes and 819 edges (Figure 4B). In this context, the 76 nodes corresponded to the 76 targets and the 819 edges represented the correlations between these targets. Following an analysis with Centiscape 2.2 software, using the criteria of greater than average Betweenness, Closeness and Degree, a total of 12 core targets were identified (Table 1, Figure 4C). The intersecting targets were ranked in ascending order based on their degree values, which included STAT3, EGFR, ESR1, PTGS2, NF-κB1, JUN, FOS, MMP9, CCND1, GSK3B, NFE2L2 and ABCG2 (degree ≥ 25). The top six targets with the highest association were STAT3, EGFR, ESR1, PTGS2, NF-κB1 and JUN, with degree values of 55, 53, 50, 50, 49 and 49, respectively.

### 2.6. GO and KEGG Enrichment Analysis

In this research, a comprehensive strategy for bioinformatics analysis was used, leveraging the DAVID v6.8 platform for a thorough multi-level functional annotation of the identified targets. This methodology was combined with the Microbiological Letter Cloud platform (www.bioinformatics.com.cn) to improve data visualization. The enrichment analysis for Gene Ontology (GO) revealed 391 categories, including 51 Cellular Components (CCs), 148 Molecular Functions (MFs) and various Biological Processes (BPs), all with a significance level set at *p* < 0.001. This analysis emphasized the top 20 biological functions that exhibited the strongest correlations (Figure 4D), such as chromatin remodeling, the negative regulation of apoptotic processes, and responses to inflammation. The CC analysis primarily focused on components like the cytoplasm, plasma membrane, and nucleus, while the MF analysis was centered around aspects including protein binding, ATP binding, and similar protein interactions. The KEGG pathway enrichment analysis uncovered 146 signaling pathways, with the leading 20 pathways being chosen based on a *p* < 0.001 significance criterion (Figure 4E). These pathways were predominantly associated with cancer, the PI3K-Akt signaling pathway, human papillomavirus infection and the MAPK signaling pathway. Although FS may influence ulcerative colitis through multiple signaling pathways [27], the KEGG pathway enrichment analysis identified 12 core targets, which were primarily enriched in the cancer, PI3K-Akt and MAPK signaling pathways, based on degree value.

### 2.7. Molecular Dynamics Simulation Results

**Figure 5:**
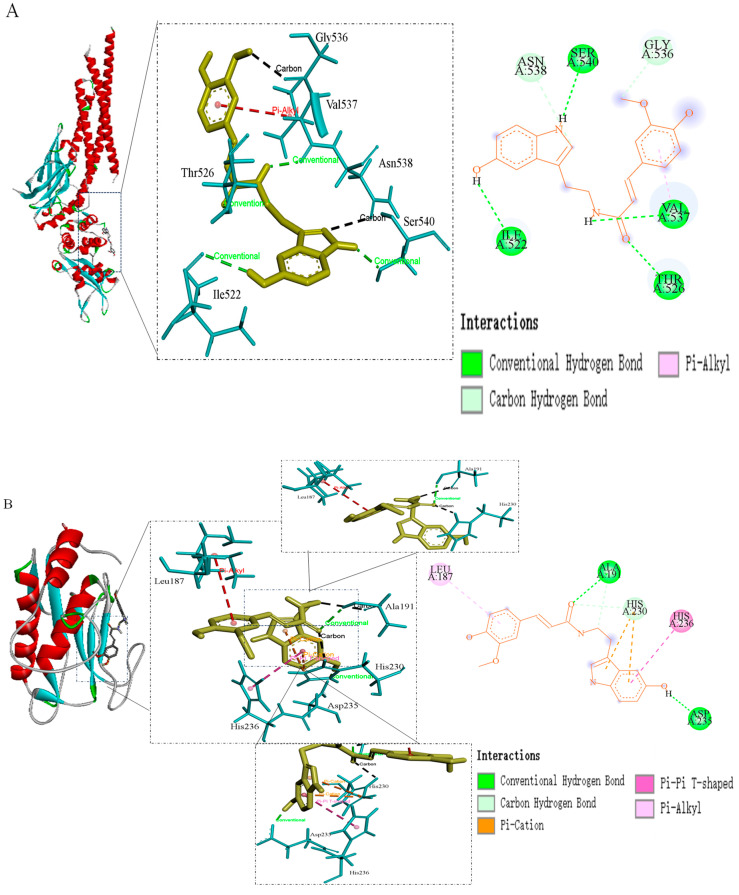

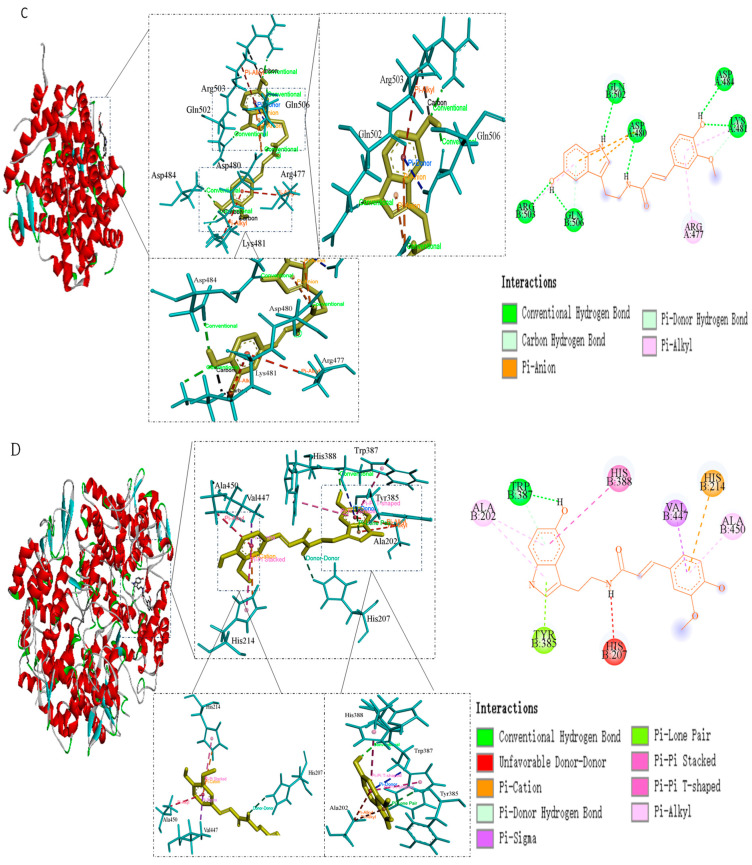

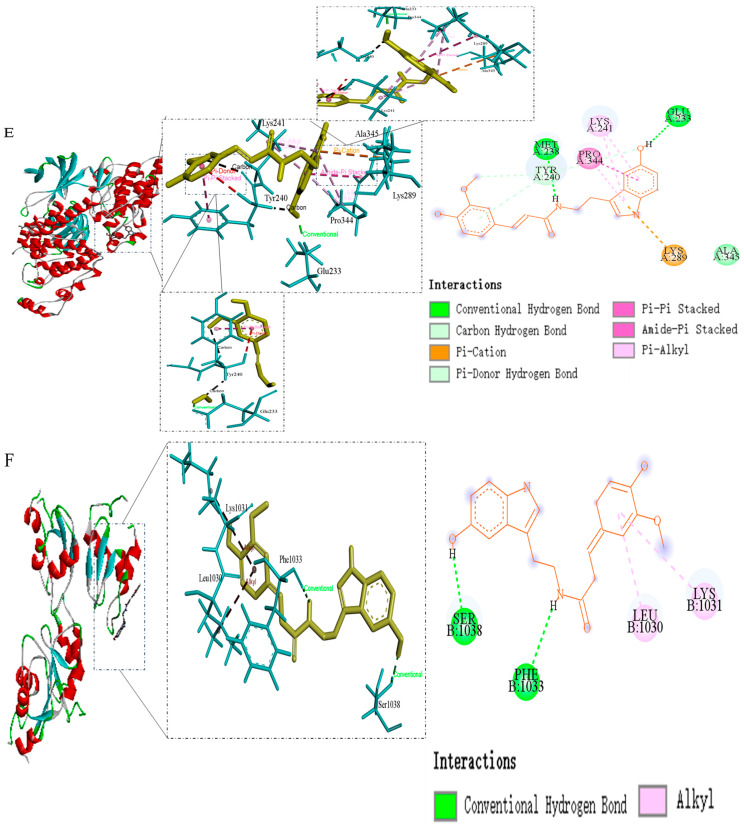
Plot of molecular docking results of FS (yellow) with STAT3 (**A**), EGFR (**B**), ESR1 (**C**), PTGS2 (**D**), JUN (**E**) and NF-κB1 (**F**) proteins (light blue).

In the PPI network analysis, the top six core targets were selected for molecular docking with FS based on their degree values to further validate the accuracy of the analysis(Figure 5). It is widely accepted that a smaller minimum binding energy in molecular docking indicates a stronger binding affinity between the molecule and the target protein [28]. This study results revealed that the docking stability of EGFR with FS was superior (−5.19 kcal/mol) (Table 2). Additionally, FS formed two hydrogen bonds with the amino acid residues Ala-191, ASP-235, and His-230 of EGFR, two cation-π bonds with His-230, one π-π bond with His-236, and one non-covalent hydrophobic bond with Leu-187.

### 2.8. Formatting of Mathematical Components

**Figure 6:**
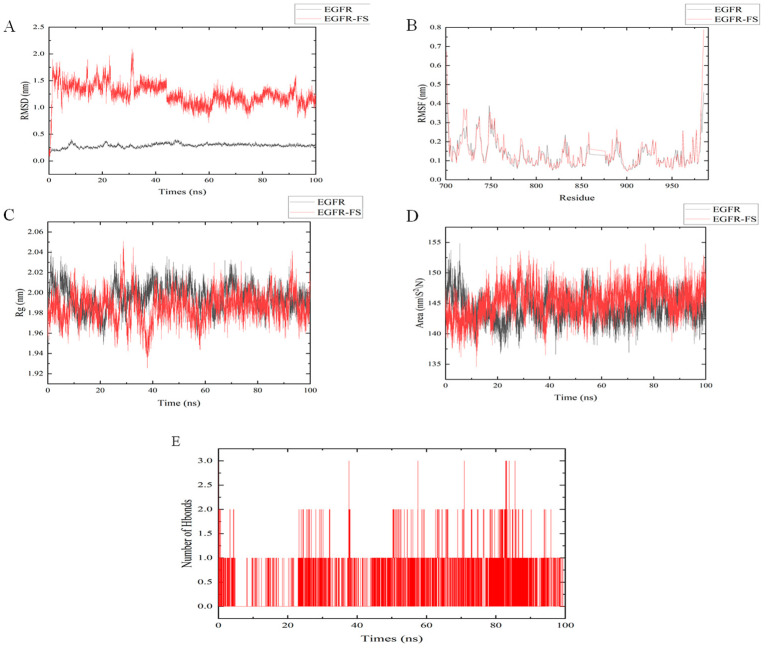
MD simulation between FS and EGFR. (**A**) RMSD results of FS in complex with EGFR within 100 ns. (**B**) RMSF results of FS in complex with EGFR. (**C**) Rg results of FS in complex with EGFR within 100 ns. (**D**) SASA results of FS in complex with EGFR within 100 ns. (**E**) Hydrogen bonding number of FS complexed with EGFR within 100 ns.

Molecular dynamics simulations of the FS and EGFR complexes were conducted using GROMACS to evaluate their interactions. The Root Mean Square Deviation (RMSD) of the FS-EGFR complex was calculated to assess global structural stability (Figure 6A). During 0–60 ns, RMSD values increased then plateaued after 60 ns, indicating system equilibration. In the same simulation, Root Mean Square Fluctuation (RMSF) analysis characterized local residue flexibility (Figure 6B). EGFR residues 720–730 showed significant fluctuations, suggesting conformational mobility in this region. The Radius of Gyration (Rg) is an indicator used to assess the overall compactness of a protein. This simulation calculated the radius of gyration between FS and EGFR EGFR (Figure 6C) and the results demonstrated that the radius of gyration of FS and EGFR decreased following the binding of FS and EGFR compared to that before binding, indicating that the binding of FS rendered EGFR more compact than before. The solvent-accessible surface area (SASA) is an index used to evaluate protein surface area. The solvent-accessible surface area between FS and EGFR was calculated in this simulation (Figure 6D) and the results indicated no significant change in the solvent-accessible surface area of EGFR before and after the binding of FS and EGFR, suggesting that ligand binding had minimal impact on the structure of EGFR. The hydrogen bonding between FS and EGFR was calculated in this simulation (Figure 6E) and the results showed that the number of hydrogen bonds varied in the range of 1–3 during the 100 ns simulation.

## 3. Materials and Methods

### 3.1. Plant Materials and Reagents

Safflower seed meal was purchased from Xinjiang Karamay Red Fruit Bio-Products Co., Ltd. (Karamay, China). Silica gel was purchased from Qingdao Ocean Chemical Co., Ltd. (Qingdao, China). Methanol and acetonitrile (HPLC grade) were purchased from Thermo Fisher Scientific Reagents, Waltham, MA, USA. The deuterium reagent was purchased from Macklin Biochemical Co., Ltd. (Shanghai, China). The NO assay kit was purchased from Biyuntian Biotechnology (Wuhan, China). A penicillin–streptomycin mixture (100X) was purchased from Solebo Technology Co., Ltd. (Beijing, China). Fetal bovine serum (FBS) and phosphate-buffered saline (PBS) were purchased from Guangzhou Hucheng Technology Co., Ltd. (Guangzhou, China). DMEM was purchased from Shanghai Darthel Biotechnology Co., Ltd. (Shanghai, China) Mouse monocyte macrophage RAW 264.7 cells were obtained from the Stem Cell Bank of the Chinese Academy of Sciences. Lipopolysaccharide (LPS, ≥99%) TRIzol^®^ reagent and 5-AminoSalicylicAcid (5-ASA, ≥99%) were purchased from Sigma-Aldrich (St. Louis, MO, USA). DEPC water was purchased from Shanghai Sangon Biotech Co., Ltd. (Shanghai, China). PerfectStart^®^ Uni RT&qPCR Kit was purchased from Beijing Quanshijin Biotechnology Co., Ltd. (Beijing, China). PerfectStart^®^ Green qPCR SuperMix was purchased from Beijing Solarbio Technology Co., Ltd. (Beijing, China).

Software: Cytoscape 3.10.2 (a popular web-based pharmacology visualization tool), Chem Bio 3D 20.0 (software for drawing chemical structures), Auto Dock Tools 1.5.7 (software for molecule-protein molecule docking), PyMOL 3.1.3, Discovery Studio 4.5 software (software for molecule as well as protein visualization software for molecules and proteins), and Gromacs 2020.6 (for molecular dynamics simulations).

### 3.2. Extraction and Purification

In total, 4 kg of dried safflower seed meal powder was subjected to an extraction process, using 95% ethanol as the solvent. This extraction was carried out under reflux conditions, which involved heating the mixture for three cycles of two hours each. The material-to-liquid ratio was maintained at 1:20 (g/mL), ensuring that the powdered material was adequately saturated with the solvent. After completing the extraction cycles, the combined filtrate was concentrated, resulting in a total ethanol extract weighing 466 g. Following this step, the ethanol extract was dispersed in seven liters of hot distilled water and underwent a series of sequential extractions with different solvents. The first solvent used was petroleum ether, which has a boiling range of 60 °C to 90 °C, followed by ethyl acetate and saturated n-butanol. Each solvent was applied three times to maximize extraction efficiency. After these extractions, vacuum concentration techniques were implemented to concentrate the extracts, yielding 158 g of petroleum ether extract, 120 g of ethyl acetate extract, and 228 g of n-butanol extract. In a subsequent phase, a portion of the ethyl acetate extract, specifically 90 g, was dissolved in methanol and thoroughly mixed with 120 g of silica gel, with a mesh size of 100–200. This mixture was placed in an evaporating dish, and the methanol was evaporated using a rotary evaporator to yield a solid sample. The prepared samples were then introduced to a silica gel column with a mesh size of 200–300 for the purpose of separation. A gradient elution was utilized in this process, employing a dichloromethane–methanol system with various ratios of solvents, specifically 200:1, 100:1, 80:1, 60:1, 40:1, 20:1, 10:1, 1:1, and 0:100 (*v*/*v*). Throughout the elution, fractions were monitored and analyzed using TLC, and fractions with similar polarities were subsequently combined, ultimately resulting in six distinct fractions.

Fr. B (22 g), underwent a meticulous process of column chromatography on 200–300 mesh silica gel. In this procedure, a mixture of dichloromethane and methanol, in volumetric ratios ranging from 60:1 to 1:1, served as the eluent. This chromatographic separation was followed by an analysis using TLC, which allowed for the identification and consolidation of fractions with similar chemical characteristics. This step culminated in the division of Fraction B into four distinct subfractions, designated as Fr. B1 through Fr. B4. In the subsequent purification phase, subfraction Fr. B2, which weighed 200 mg, was processed further using Sephadex LH-20 and RP-HPLC. The mobile phases for this intricate purification involved both pure methanol and a gradient involving methanol and water—specifically, transitions from a 25:75 to a 70:30 ratio (*v*/*v*) at a flow rate of 3 mL/min. This careful optimization ultimately succeeded in isolating compound **2**, which was obtained in an amount of 10.5 mg. Simultaneously, subfraction Fr. B3, weighing 180 mg, also underwent purification via RP-HPLC, utilizing a methanol–water gradient as the mobile phase. The flow rate for this process was adjusted to 2 mL/min, and the gradient ranged from a 50:50 to a 70:30 ratio (*v*/*v*). This technique effectively led to the isolation of three distinct compounds: compound **3**, which yielded 62.7 mg; compound **5**, arriving at 8.1 mg; and compound **11**, with a final mass of 5.5 mg. Additionally, Fr. C, (7.3 g) was separated using a similar approach through column chromatography on 200–300 mesh silica gel, with a dichloromethane–methanol eluent mixture specified at a ratio of 40:1 to 1:1 (*v*/*v*). Following this separation, TLC analysis was again employed, leading to the merging of similar fractions and resulting in the formation of three new subfractions, labeled Fr. C1 to Fr. C3. The subfraction designated as Fr. C1, weighing 267.3 mg, was subjected to a purification process that involved both Sephadex LH-20 and RP-HPLC. During this procedure, methanol and a methanol–water gradient were utilized as the mobile phases, operating at a flow rate of 3 mL/min and within a composition range of 30:70 to 55:45 (*v*/*v*). This meticulous purification effort resulted in the successful recovery of several compounds: compound **1** (20.7 mg), compound **4** (70.9 mg), compound **6** (10.8 mg), and compound **7** (11.2 mg). For the subsequent fraction, labeled as Fraction D and weighing 1.7 g, separation was accomplished through column chromatography, employing a silica gel with a mesh size of 200–300. This procedure utilized a solution of dichloromethane and methanol in a volumetric ratio of 10:1 to 1:1 as the mobile phase. Following the TLC analysis, similar fractions were merged, resulting in the formation of three distinct subfractions, referred to as Fr. D1 through D3. The purification of the subfraction Fr. D1, which weighed 106.3 mg, was achieved using both Sephadex LH-20 and RP-HPLC techniques. Here, pure methanol and a methanol–water gradient were re-employed as the mobile phases, flowing at a rate of 4 mL/min and ranging from a ratio of 30:70 to 90:10 (*v*/*v*), ultimately leading to the isolation of compound **8** (12.1 mg). Additionally, Fraction E, with a total weight of 4.3 g, underwent separation via column chromatography, again using a silica gel of 200–300 mesh size. This step was facilitated by an elution of dichloromethane–methanol in a ratio varying from 5:1 to 0:1 (*v*/*v*). After conducting TLC analysis to evaluate the fractions, similar fractions were combined to yield four new subfractions, labeled Fr. E1 through E4. The purification of subfraction Fr. E1, which had a mass of 221.3 mg, was executed using both Sephadex LH-20 and RP-HPLC. In this case, pure methanol and a methanol–water gradient (flow rate of 2 mL/min; ranging from 40:60 to 77:33, *v*/*v*) served as the mobile phases, culminating in the successful isolation of compounds **9** (4.1 mg) and **10** (3.6 mg).

### 3.3. Cell Culture

RAW264.7 cells were seeded in complete DMEM medium, which contained 1% penicillin–streptomycin (100 U/mL penicillin and 100 µg/mL streptomycin) and 10% FBS and were cultured in a 37 °C incubator with 5% CO_2_ for 24 h. The medium was changed daily. When the cell density reached approximately 80%, the cells were passaged. All experiments were conducted using cells in the logarithmic growth phase. 5-ASA and each compound were dissolved in a small amount of DMSO and then diluted with DMEM medium to achieve final concentrations of 0, 25, 50, 100, and 200 μg/mL. The cells were divided into four groups: a control group, an LPS model group, a 5-ASA positive drug group, and a treatment group. The control group was cultured in DMEM medium, the model group was treated with 1 μg/mL LPS, and the treatment group was administered each compound as described above.

The impact of serotonin derivatives on the viability of RAW264.7 cells was evaluated using the CCK-8 assay [29]. RAW264.7 cells in the logarithmic growth phase were seeded at a density of 2 × 10^4^ cells per well in a 96-well plate. The plate was incubated at 37 °C in a 5% CO_2_ atmosphere for 24 h to allow for cell adhesion. Following this incubation, the original culture medium was discarded. Subsequently, 5-ASA-positive drugs at concentrations of 0, 25, 50, 100, and 200 μg/mL, along with each test compound, were added to the corresponding wells at a volume of 200 μL per well. After the addition of samples, the 96-well plate was returned to the 37 °C, 5% CO_2_ incubator for an additional 24 h. After this incubation, 10 μL of CCK-8 solution was added to each well, and the incubation continued at 37 °C for 1 h. After incubation, the plate was removed, and absorbance (OD) was measured at 450 nm to determine cell viability. The formula for calculating cell viability is as follows: Cell viability (%) = [A(treatment) − A(blank)]/[A(control) − A(blank)] × 100%.

### 3.4. Anti-Inflammatory Effect of Serotonin Derivatives In Vivo

The amount of NO in the cell liquids was measured using the Griess method [26]. Total cells grew rapidly, sown in 96-well plates, and each deepening layer contained 20,000 cells. They were incubated at 37 °C for 24 h in 5% atmosphere at 37 °C to allow cell adhesion. Following this incubation, the original medium was discarded, and 100 μL of incomplete DMEM medium was introduced to each well to induce a starvation period of 8 h. DMEM was then in the positive or negative control group. Then, 200 μL of media containing 1 μg/mL LPS was introduced. After 24 h of incubation at 37 °C under 5% CO_2_, 50 μL of liquid from each well was transferred to a new 96-well plate. To each well, 50 μL of Griess Reagent I and 50 μL of Griess Reagent II were added and thoroughly mixed. Finally, the absorbance was measured at a wavelength of 540 nm, allowing for the calculation of the nitric oxide concentration.

Real-time quantitative polymerase chain reaction (RT-qPCR) was employed to evaluate the mRNA levels of pro-inflammatory cytokines (IL-1β, IL-6 and TNF-α) and the anti-inflammatory cytokine (IL-10) in RAW 264.7 cells [30]. RAW 264.7 cells were seeded in 6-well plates, with a density of 2 × 10^5^ cells per well, and allowed to incubate for a duration of 24 h. After this initial incubation, the existing medium was completely removed to initiate a process of starvation. This was achieved by adding 1 mL of incomplete DMEM to each well, which was maintained for an additional 8 h period. Once this starvation phase had concluded, the incomplete medium was discarded, and each well was treated with 200 mL of a solution containing 1 μg/mL of LPS. In this setup, various groups were established, including a blank control, positive control, and drug treatment groups. Following a thorough 24 h incubation period after LPS treatment, Total RNA extraction and cDNA synthesis were performed using TRIzol^®^ reagent (Sigma-Aldrich) in an RNase-free environment. The specific steps are as follows: First, the culture medium was removed, and the cells were washed three times with PBS. Next, 500 μL of TRIzol^®^ was added to lyse the cells, and the lysate was scraped and transferred to a centrifuge tube. The mixture was vortexed and incubated on ice for 5 min. Subsequently, 0.4 mL of chloroform was added, followed by vortexing to mix and incubation on ice for 3 min. The mixture was then centrifuged at 12 × 10^3^ rpm at 4 °C for 15 min. After centrifugation, 0.5 mL of isopropanol was added to the supernatant, which was incubated on ice for 1 h before centrifugation at 12 × 10^3^ rpm at 4 °C for 10 min. The precipitate was washed twice with 1 mL of 75% DEPC-ethanol. After drying the precipitate, it was dissolved in 30 μL of RNase-free water. RNA purity was verified using a nucleic acid analyzer (Quawell Q5000, San Diego, CA, USA) with an A260/A280 ratio of 1.8–2.1, and the concentration was adjusted uniformly to 40 ng/μL. In total, 2 μL of RNA was used with the PerfectStart^®^ Uni RT&qPCR Kit (Beijing, China) to synthesize cDNA: the 20 μL reaction mixture was incubated at 42 °C for 15 min, followed by inactivation at 85 °C for 5 s. The cDNA concentration was adjusted to 40 ng/μL with TE buffer, aliquoted, and stored at −20 °C to avoid repeated freeze–thaw cycles. For qPCR amplification, two-step amplification was performed using PerfectStart^®^ Green qPCR SuperMix (Beijing, China). The reaction mixture was prepared according to the manufacturer’s instructions, with the following cycling program: 94 °C for 30 s for pre-denaturation, followed by 45 cycles of 94 °C for 5 s and 60 °C for 30 s. The mRNA expression levels of IL-1β, IL-6, TNF-α and IL-10 were determined using SYBR Green fluorescence quantitative PCR and the relative mRNA expression was calculated using the 2^−ΔΔCt^ method [31]. The primers used were provided by Bioengineering Biologicals and the specific primer sequences are listed in Table 3 [18].

### 3.5. Compound Targets, Ulcerative Colitis Target Acquisition and Intersecting Genes

To explore the role of serotonin derivatives in the treatment of ulcerative colitis, the researchers selected N-feruloyl serotonin (FS) for analysis through online drug platforms. They utilized the Swiss Target Prediction Database along with the SEA Database, focusing on “Homo sapiens” for their target screening process. After eliminating duplicates, they aimed to identify the appropriate targets. Subsequently, they accessed the CTD Database and the GeneCards Database to locate targets associated with ulcerative colitis, using “Ulcerative colitis” as the key search phrase. They established correlation scores of 22.75 and 4.38 to filter the outcomes and further removed any duplicates to isolate disease-related targets. Finally, they employed Venny 2.1.0 software to identify shared targets between FS and ulcerative colitis.

### 3.6. Screening of Core Targets and Construction of Compound–Target Networks

The researchers created maps of protein–protein interaction by using the STRING database online. They focused on human proteins and kept other settings as default. The results were downloaded in “TSV” format and opened in Cytoscape 3.10.2. With CentiScaPe 2.2 software, they calculated degree value, mediator centrality and proximity to centrality. Important targets were found and studied using Cytoscape 3.10.2, which also helped build the “compound–target–disease” network.

### 3.7. GO and KEGG Enrichment Assay Experiments

The researchers used the DAVID database to study the overlap between disease and compound target genes. They set the identifier to “OFFICIAL_GENE_SYMBOL”, chose “gene list” as the list type and specified “Homo sapiens” as the species. They performed GO and KEGG analyses, considering results significant if *p* < 0.01. Smaller *p* values meant stronger links. They identified the top 20 biological processes, molecular functions, cellular components and signaling pathways. They also created GO information bar and pathway bubble graphs using a microbiology platform.

### 3.8. Molecular Docking

The 3D structures of the main targets were downloaded from the RCSB PDB website in PDB format. The 3D structures of the small molecules were made using Chem Draw version 20.0 and saved as mol2 files. Large protein targets were processed using PyMOL version 3.1.3 to remove hydrogen and ligands. Both large proteins and small molecules were prepared for hydrogenation, charge calculation and atom type determination using Auto Dock Tools version 1.5.7 and then saved as pdbqt files. The compounds were checked through molecular docking with the main target and the results were viewed using Discovery Studio version 4.5.

### 3.9. Molecular Dynamics Simulation

Utilizing molecular docking energy parameters to optimize potential binding targets, a molecular dynamics simulation study was conducted using the Gromacs 2020.6 computing platform. The topology of the complex system was constructed. The CHARMM 36 force field was employed for atomic parameterisation and the TIP3P three-point water molecule model was used to construct the solvent environment of the system. The system was solvated using a cubic periodic boundary water box and sodium ions were added to maintain charge balance. During the energy optimization stage, the steepest descent algorithm was configured to perform 50,000 steps of energy minimization calculations to eliminate spatial potential resistance. The kinetic simulation was executed in stages: (1) the NVT system pre-equilibration stage, employing the Verlet integral algorithm combined with the PME electrostatic force correction method at a constant temperature of 300 K for 1 ns pre-relaxation, and (2) the NPT system equilibrium stage, utilizing the Parrinello–Rahman pressure coupling method to maintain a constant pressure environment of 1 bar for a duration of 1 ns. The formal simulation phase was implemented with 100 ns of productive operations, with the time step set to 2 fs, and 5 × 10^4^ steps of trajectory sampling were accumulated. Parameter transfer and parallel computation were realized through the chained commands of the gmx module throughout the process. The gmx analysis suite was employed in the post-processing stage of the trajectory for multidimensional conformational evaluation. The protein and complex conformational shift was quantified by Root Mean Square Deviation (RMSD) and analyzed by Root Mean Square Fluctuation (RMSF). Amino acid residue flexibility was characterized by RMSF, structural density evolution was evaluated by Radius of Gyration (Rg) and hydrophobic core exposure was monitored by the solvent-accessible surface area (SASA). Simultaneous statistics on the dynamic formation of intermolecular hydrogen bonds were used to construct a comprehensive evaluation model for the stability of the composite system.

### 3.10. Statistical Analysis

Data recording, analysis, processing, and graphing were conducted using various software tools, specifically Microsoft Excel 2021, GraphPad Prism 9.0, and Origin Pro 2021. To assess the differences between groups, a one-way analysis of variance (ANOVA) was employed. The results of all experiments are presented as mean values along with the standard error of the mean, expressed as Mean ± SEM. In this study, statistical significance was determined at a threshold of *p* < 0.05, indicating that any *p*-values below this level were considered statistically meaningful.

## 4. Discussion

Safflower is a versatile cash crop with extensive distribution across China, Morocco, Egypt, India and other countries [32]. The blossoms of this particular plant are commonly utilized in traditional medicinal practices for the treatment of various health issues. These include gynecological conditions [33], cardiovascular diseases and cerebrovascular disorders [34], along with other ailments such as blood stasis [35] and osteoporosis [36]. The therapeutic applications of the flowers highlight their importance in holistic health approaches. Furthermore, researchers have successfully isolated and identified more than 200 distinct compounds from this genus [37]. Among them, serotonin derivatives have been primarily extracted from safflower seeds and their meal, indicating a significant potential for pharmacological applications and requiring further study into their health benefits [38]. Approximately 20 of these compounds demonstrate anticoagulant, hepatoprotective, neuroprotective, and antioxidant properties [25,39]. In the present study, 11 serotonin derivatives were isolated and identified from safflower seed meal using a systematic compositional isolation method. Notably, N-trans-feruloyl-3,5-dihydroxyindolin-2-one and Bufoserotonin A were isolated from safflower seed meal for the first time.

Research has demonstrated that ulcerative colitis onset is frequently associated with aberrant NO levels and the disrupted expression of inflammatory mediators [40]. As a result, this research utilized 11 serotonin derivatives to assess the effect of NO on LPS-stimulated RAW 264.7 cells and its role in regulating crucial cytokines linked to ulcerative colitis, specifically IL-6, TNF-α, IL-1β, and IL-10, through Griess and RT-qPCR assays. The results revealed that compounds **3**, **4**, **5**, **6**, **7**, and **10** showed inhibitory activities on NO, leading to a reduction in IL-6, TNF-α, and IL-1β while increasing the expression of IL-10. Among these, FS showed the most significant effects.

In this study, we investigated the potential targets and signaling pathways of FS in the treatment of ulcerative colitis using network pharmacology, molecular docking and molecular dynamics simulations. The findings indicated that FS could modulate the progression of ulcerative colitis via multiple targets, with STAT3, EGFR, ESR1, PTGS2, NF-κB1 and JUN identified as the six key targets significantly involved in the pathogenesis and progression of ulcerative colitis. GO and KEGG enrichment analyses suggested that FS could exert its anti-ulcerative colitis effects through the integration of related signaling pathways, such as PI3K/Akt and MAPK, alongside other associated pathways to mediate its anti-inflammatory effects in ulcerative colitis. Molecular docking generally posits that the docking results of compounds and target proteins are more favorable when the binding energy is less than −5 kcal/mol, with lower binding energies indicating higher affinity and a greater likelihood of functional interaction. The molecular docking results demonstrated that the binding energies of FS with the key targets EGFR and ESR1 were both below −5.0 kcal/mol, with the lowest binding energy observed between FS and EGFR. Furthermore, to substantiate the stability of the complexes and provide theoretical support for their potential biological roles, we conducted a 100 ns molecular dynamics simulation analysis of the EGFR-FS complex system. The RMSD results indicated more stable binding, while the RMSF revealed the significant flexibility of structural domains between EGFR-FS and the smaller Rg suggested a more compact structure post binding, with numerous hydrogen bonds present between the complexes. It has been established that in ulcerative colitis, the expression level of EGFR is significantly elevated [41] and EGFR is critically involved in the activation of PI3K and AKT. The regulation and release of pro-inflammatory cytokines are influenced by the PI3K/AKT signaling pathway, where p-AKT subsequently stimulates the downstream NF-κB signaling pathway, consequently enhancing the expression and secretion of pro-inflammatory cytokines. This leads to an imbalance in cytokine secretion, a cascade of inflammatory responses, and mucosal injury and affects the progression of ulcerative colitis [42]. Therefore, FS may influence the onset and progression of ulcerative colitis through its interaction with the EGFR.

## 5. Conclusions

In this research, a total of 11 serotonin derivatives were extracted and characterized from safflower seed meal through systematic extraction and separation methodologies. Importantly, N-trans-feruloyl-3,5-dihydroxyindolin-2-one and Bufoserotonin A were identified from safflower seed meal for the first time. Serotonin derivatives **1**, **3**, **4**, **5**, **6**, **7**, and **10** displayed notable anti-inflammatory properties in an LPS-induced macrophage inflammation model, with N-feruloyl serotonin showing the most significant effect. Additionally, a combination of network pharmacology, molecular docking, and molecular dynamics simulations was applied to explore the anti-inflammatory impacts of these compounds by targeting essential proteins like EGFR and STAT3, as well as influencing crucial signaling pathways, including the PI3K-Akt and MAPK pathways. This research lays the groundwork for understanding the serotonin derivatives derived from safflower seed meal and clarifies their mechanisms of action, thus promoting the further exploration and application of safflower seed meal.

## Figures and Tables

**Table 1:** Information on the top 12 targets in the PPI network.

NO.	Name	Uniprot ID	Inference Score	Betweenness Centrality	Closeness Centrality	Degree
1	STAT3	P40763	66.06	357.4028265	0.0107527	55
2	EGFR	P00533	29.04	413.4285767	0.0104167	53
3	ESR1	P03372	27.54	360.2069451	0.010101	50
4	PTGS2	P35354	75.77	454.1053214	0.010101	50
5	NFKB1	P19838	58.21	259.5625728	0.010101	49
6	JUN	P05412	49.01	260.1712984	0.009901	49
7	FOS	P01100	40.92	350.4217096	0.0093458	43
8	MMP9	P14780	53.2	95.65275435	0.0092593	42
9	CCND1	P24385	44.95	92.37872806	0.0092593	41
10	GSK3B	P49841	52.59	120.5941294	0.0091743	39
11	MTOR	P42345	56.1	52.77919749	0.0088496	38
12	BCL2L1	Q07817	38.14	31.33862389	0.0086957	35

**Table 2:** Molecular binding energies of FS to targets (kcal/mol).

Acceptor	Bond Energy (kcal/mol)	Amino Acid Residue
STAT3	−3.61	Gly-536, Val-537, Asn-538, Ser-540, Thr-526, Ile-522
EGFR	−5.19	Leu-187, His-236, Ala-191, His-230, ASP-235
ESR1	−5.17	Asp-484, Gln-502, Lys-481, Asp-480, Arg-477, Gln-506, Arg-503
PTGS2	−4.65	Ala-202, Ala-450, Tyr-385, Trp-387, His-207, His-388, His-214
JUN	−4.83	Ala-345, Lys-241, Tyr-240, Pro-344, Lys-289, Glu-233, Met-238
NF-κB1	−4.35	Leu-1030, Lys-1031, Phe-1033, Ser-1038

**Table 3:** q-PCR primer sequences (species origin: Mus musculus).

Genomics	Pre-Primer (5′-3′)	Posterior Primers (3′-5′)
IL-6	TAGTCCTTCCTACCCCAATTTCC	TTGGTCCTTAGCCACTCCTTC
IL-1β	GCAACTGTTCCTGAACTCAACTT	ATCTTTTGGGGTCCGTCAACT
TNF-α	CCCTCACACTCAGATCATCTTCT	GCTACGACGTGGGCTACAG
IL-10	GCTCTTACTGACTGGCATGAG	CGCAGCTCTAGGAGCATGTG
GAPDH	CATCACTGCCACCCAGAAGACTG	ATGCCAGTGAGCTTCCCGTTCAG

## Data Availability

The data and results presented in this study are available upon request from the first and corresponding authors.

## Supplementary Materials

The following supporting information can be downloaded at: https://www.mdpi.com/article/10.3390/molecules30132886/s1, Figure S1. ^1^H-NMR spectrum of Compound **1**. Figure S2. ^13^C-NMR spectrum of Compound **1**. Figure S3. ^1^H-NMR spectrum of Compound **2**. Figure S4. ^13^C-NMR spectrum of Compound **2**. Figure S5. NOESY spectrum of Compound **2**. Figure S6. HSQC spectrum of Compound **2**. Figure S7. HMBC spectrum of Compound **2**. Figure S8. H-H COSY spectrum of Compound **2**. PCR primer sequences (species origin Mus musculus).
