# Supplementary material for: Revealing Serotonin Derivatives in Safflower Seed Meal as Potential Anti-Ulcerative Colitis Drugs: In Vitro and Computational Evidence
Source: Molecules. 2025 Jul 7;30(13):2886. doi: 10.3390/molecules30132886 (PMC12251380; doi:10.3390/molecules30132886)
Supplement: Supplementary file 1 [file molecules-30-02886-s001.zip › Supporting Information Figures S1-S8.pdf]

Revealing Serotonin Derivatives in Safflower Seed Meal as Potential Anti-Ulcerative Colitis Drugs: In Vitro and Computational Evidence

Liang Zhang, Md Hasan Ali, Chao Jiang, Furong Fan, Furong Zhu, Yating Lu, Mengwei Jia, Haipeng Yin, Jianwang Wei, Dongsen Wu, Shenghui Chu and Min Liu

List of Supporting Information

Figure S1. ^{1}H-NMR spectrum of Compound **1**
Figure S2. ^{13}C-NMR spectrum of Compound **1**
Figure S3. ^{1}H-NMR spectrum of Compound **2**
Figure S4. ^{13}C-NMR spectrum of Compound **2**
Figure S5. NOESY spectrum of Compound **2**
Figure S6. HSQC spectrum of Compound **2**
Figure S7. HMBC spectrum of Compound **2**
Figure S8. H-H COSY spectrum of compound **2**.

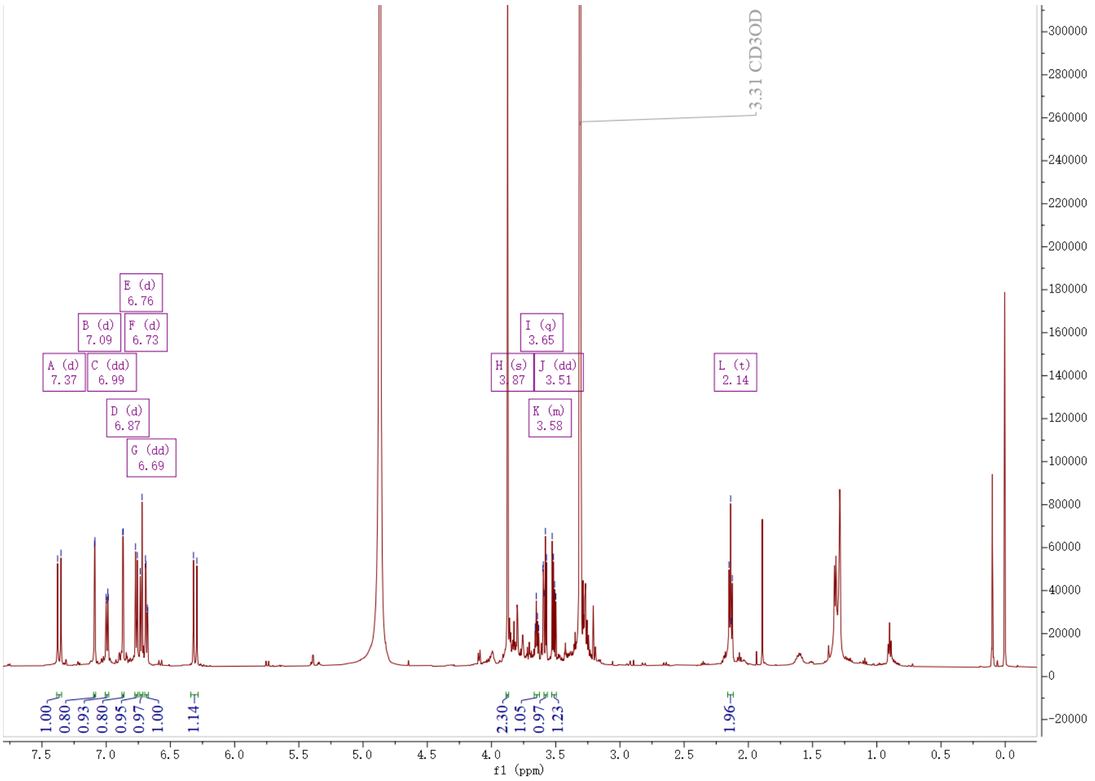

Figure S1. ^{1}H-NMR spectrum of compound **1**.

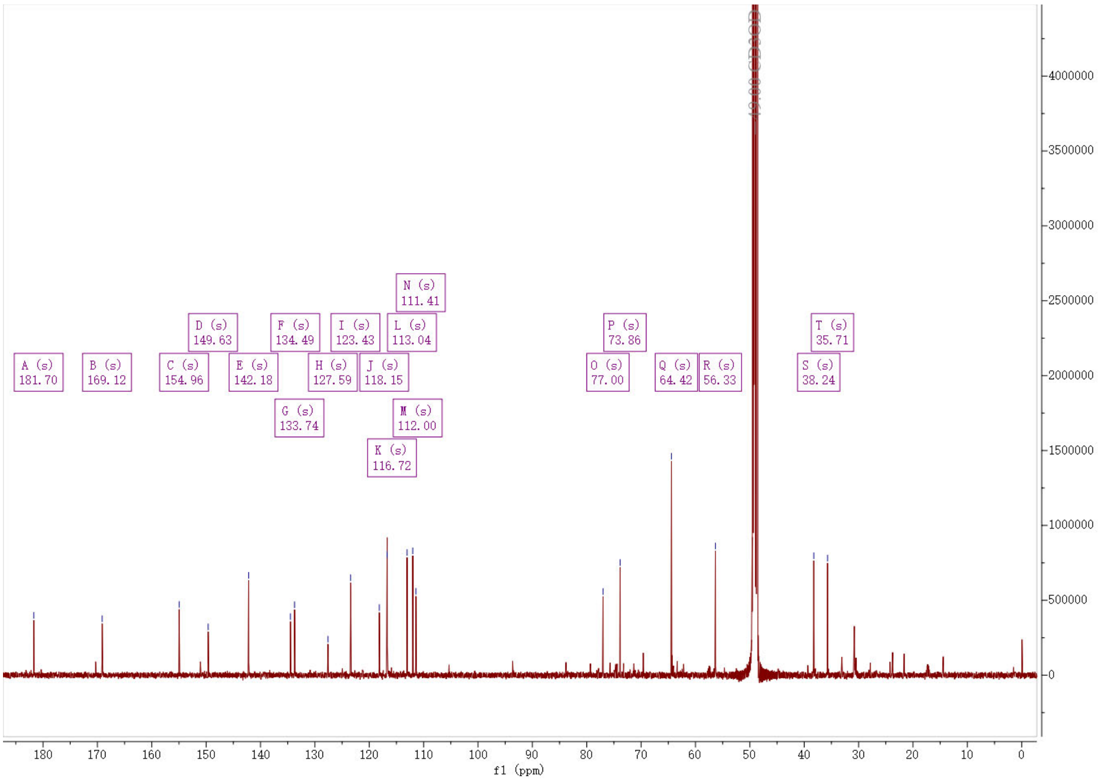

Figure S2. ^{13}C-NMR spectrum of compound **1**.

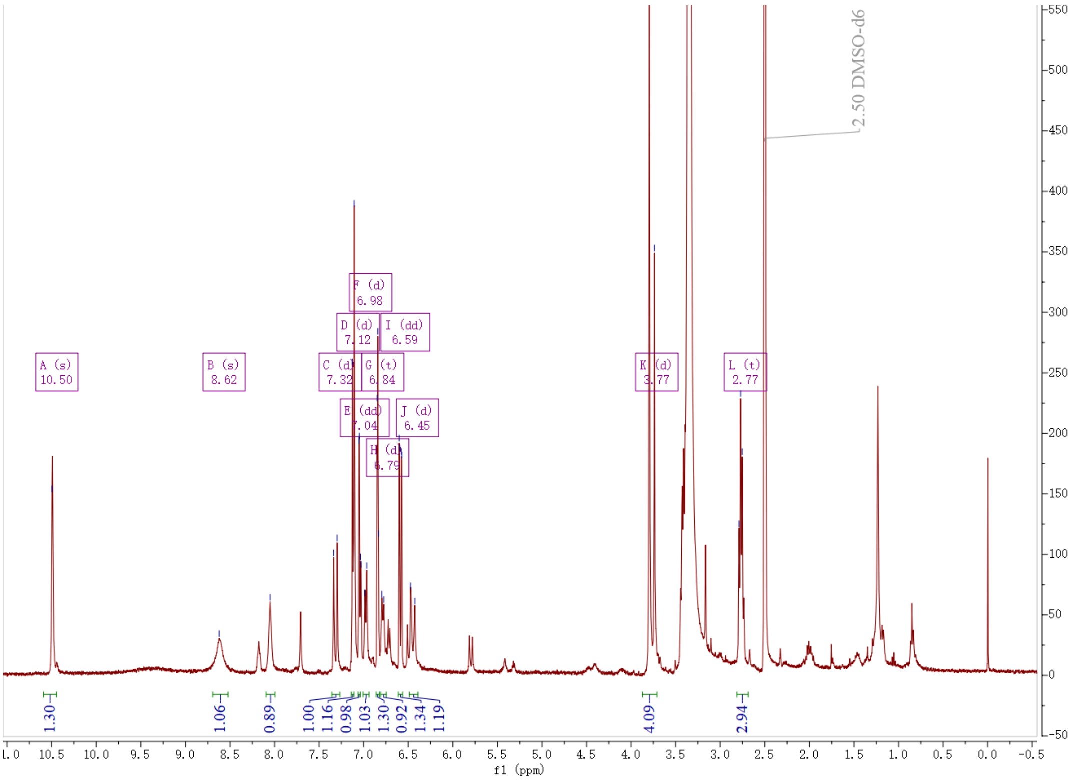

Figure S3. ^{1}H-NMR spectrum of compound **2**.

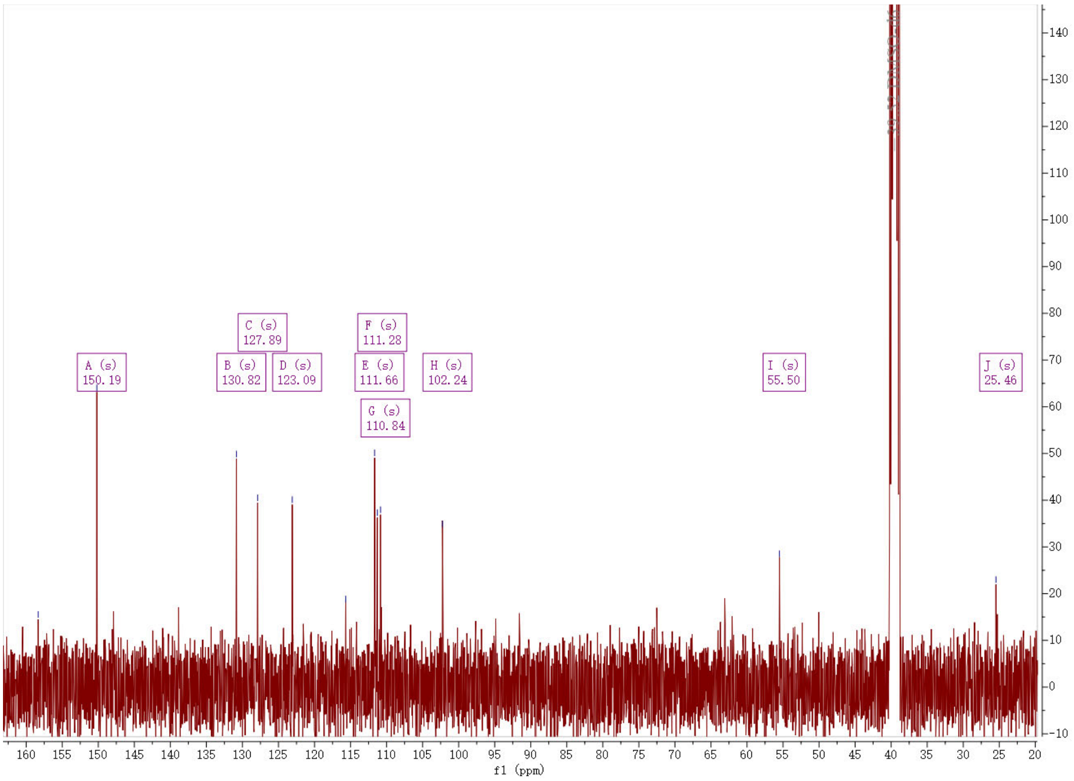

Figure S4. ^{13}C-NMR spectrum of compound **2**.

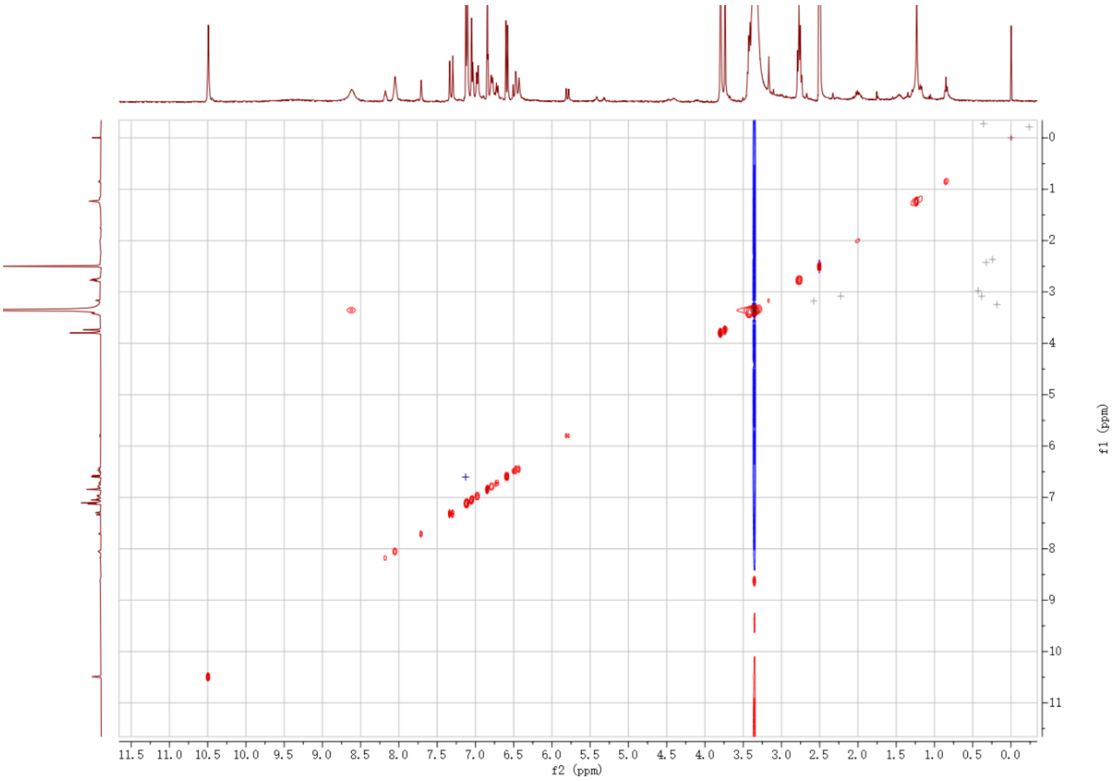

Figure S5. NOESY spectrum of compound **2**.

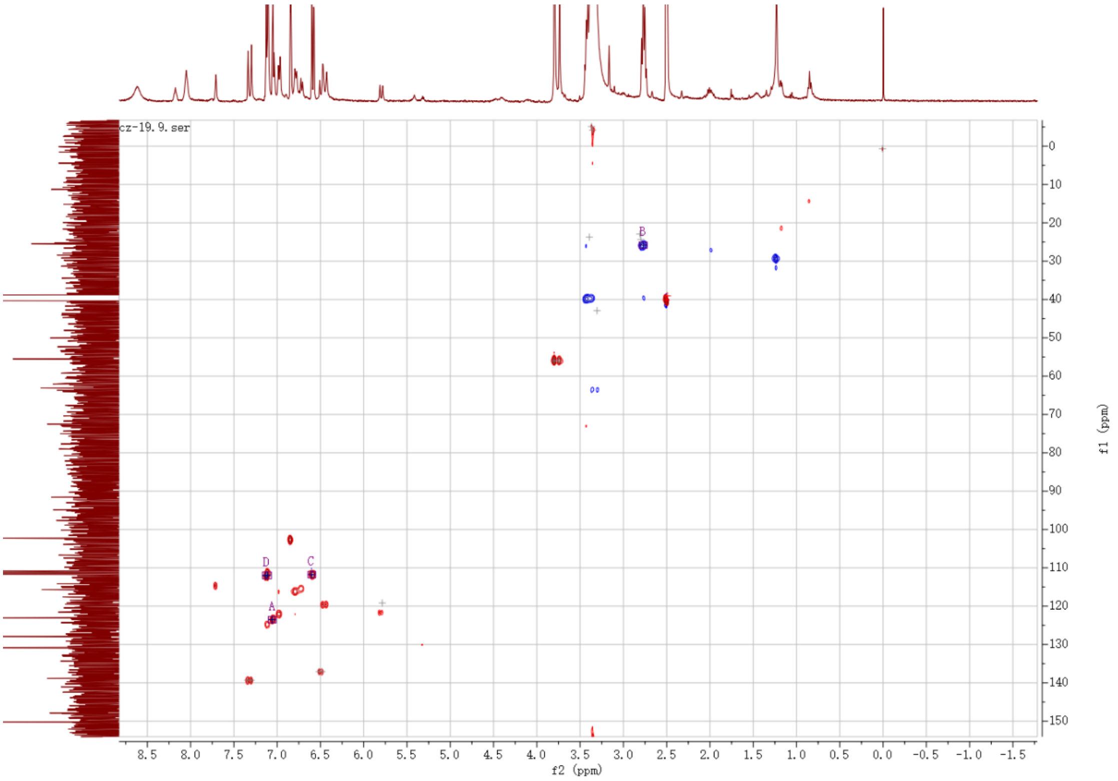

Figure S6. HSQC spectrum of compound **2**.

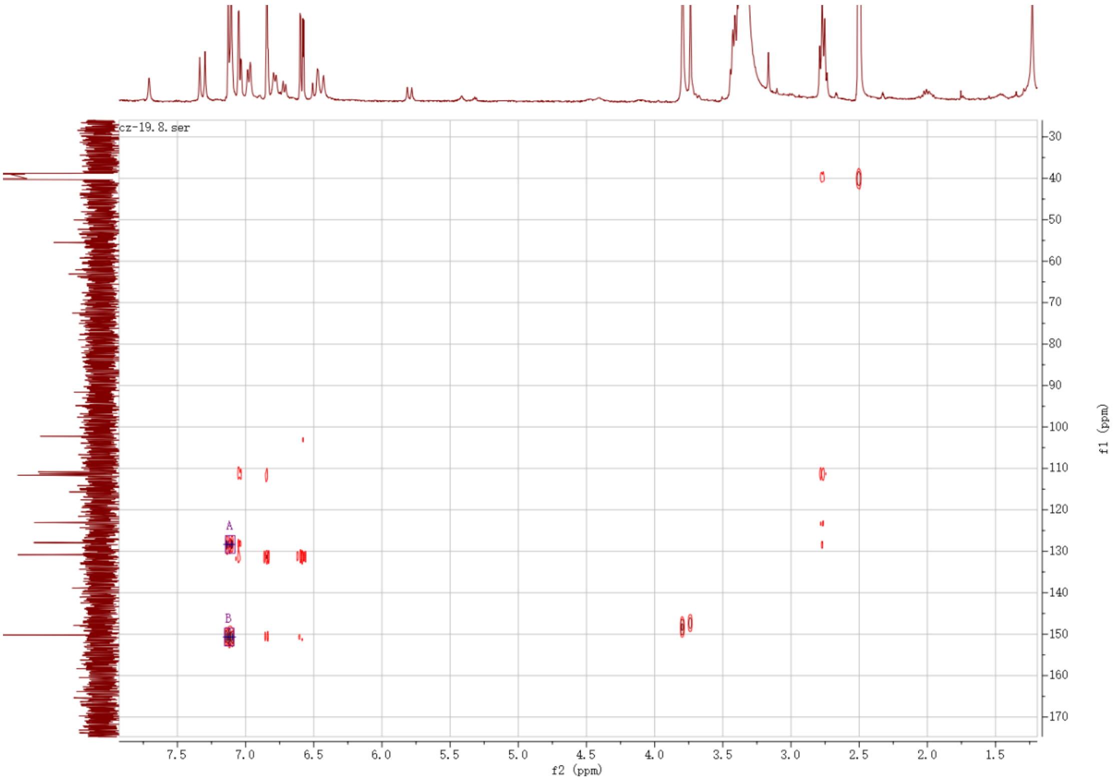

Figure S7. HMBC spectrum of compound **2**.

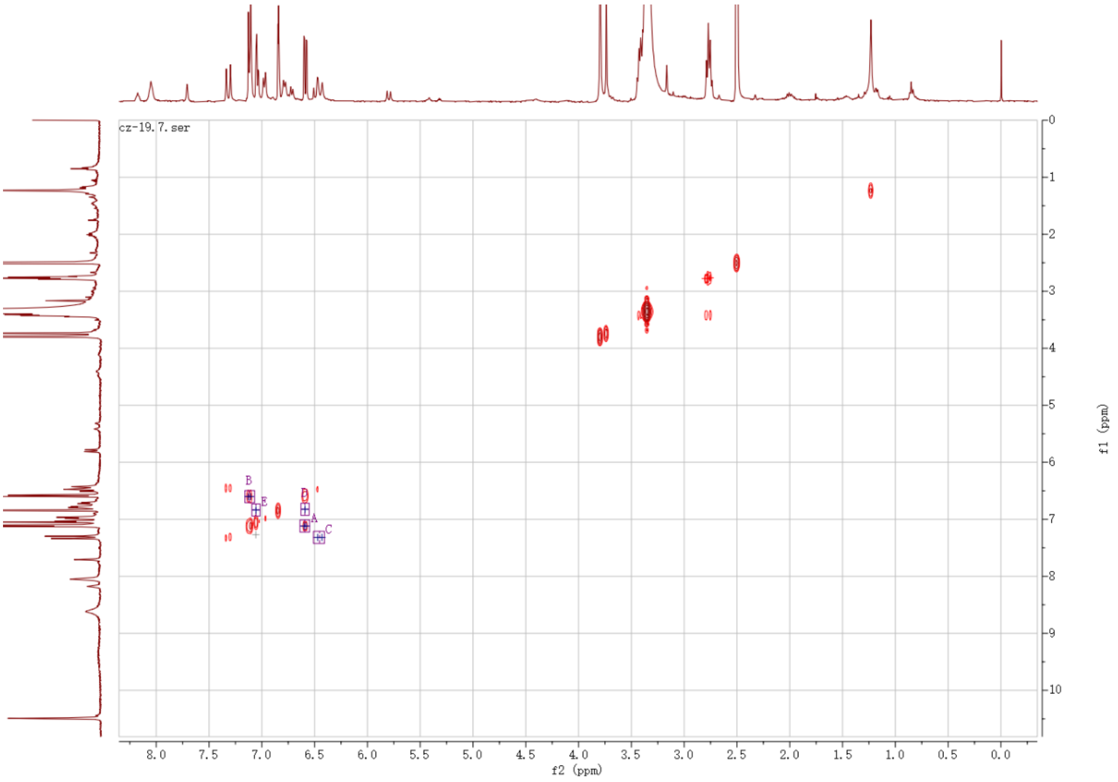

Figure S8. H-H COSY spectrum of compound **2**.
